# Development and Validation of a LASSO Prediction Model for Better Identification of Ischemic Stroke: A Case-Control Study in China

**DOI:** 10.3389/fnagi.2021.630437

**Published:** 2021-07-08

**Authors:** Zirui Meng, Minjin Wang, Shuo Guo, Yanbing Zhou, Mingxue Zheng, Miaonan Liu, Yongyu Chen, Zhumiao Yang, Bi Zhao, Binwu Ying

**Affiliations:** ^1^Department of Laboratory Medicine, West China Hospital, Sichuan University, Chengdu, China; ^2^Department of Neurology, West China Hospital, Sichuan University, Chengdu, China

**Keywords:** ischemic stroke, prediction model, laboratory variables, demographic variables, least absolute shrinkage and selection operator, smartphone app

## Abstract

**Background:**

Timely diagnosis of ischemic stroke (IS) in the acute phase is extremely vital to achieve proper treatment and good prognosis. In this study, we developed a novel prediction model based on the easily obtained information at initial inspection to assist in the early identification of IS.

**Methods:**

A total of 627 patients with IS and other intracranial hemorrhagic diseases from March 2017 to June 2018 were retrospectively enrolled in the derivation cohort. Based on their demographic information and initial laboratory examination results, the prediction model was constructed. The least absolute shrinkage and selection operator algorithm was used to select the important variables to form a laboratory panel. Combined with the demographic variables, multivariate logistic regression was performed for modeling, and the model was encapsulated within a visual and operable smartphone application. The performance of the model was evaluated on an independent validation cohort, formed by 304 prospectively enrolled patients from June 2018 to May 2019, by means of the area under the curve (AUC) and calibration.

**Results:**

The prediction model showed good discrimination (AUC = 0.916, cut-off = 0.577), calibration, and clinical availability. The performance was reconfirmed in the more complex emergency department. It was encapsulated as the Stroke Diagnosis Aid app for smartphones. The user can obtain the identification result by entering the values of the variables in the graphical user interface of the application.

**Conclusion:**

The prediction model based on laboratory and demographic variables could serve as a favorable supplementary tool to facilitate complex, time-critical acute stroke identification.

## Introduction

Stroke is currently the second cause of death worldwide and the leading cause of death in China. Approximately 70% of all strokes are ischemic and this will significantly increase the health burden due to the aging population (Zhou et al., 2016; GBD 2016 Causes of Death Collaborators, 2017; Wang et al., 2017). When it comes to treatment, intravenous tPA (tissue-type plasminogen activator) has been used to treat most acute ischemic strokes (AISs). However, it is highly risky and can be lethal in the case of intracerebral hemorrhage (ICH) (Zerna et al., 2018). Therefore, determining the stroke subtype in an early, timely, and accurate manner is essential to achieve proper treatment and good prognosis (Hankey, 2017; Deboevere et al., 2019). Furthermore, stroke mimics, which present with an acute neurological deficit simulating AIS and represent a significant percentage of all acute stroke hospital admissions, pose a diagnostic challenge to emergency physicians (Vilela, 2017; Liberman et al., 2019). A recent meta-analysis was performed on 23 studies, including a total of 15,721 patients and reported that the initial diagnosis was misdiagnosed in 26–40% of the cases. Besides, 2–26% of ischemic stroke (IS) patients were misdiagnosed (Tarnutzer et al., 2017). This might be due to the absence of acute ischemic signs or the presence of non-specific stroke symptoms on initial computed tomography (CT) imaging, as well as the interference of stroke mimics (Walsh, 2019). In such cases, the diagnosis may not be confirmed until additional imaging tests are performed several hours or even a day later, which results in missing the optimal intervention time (Martins et al., 2020). In addition, neuroimaging examination needs to be performed in a qualified medical institution with specialized equipment and under the guidance of professional physicians; these conditions seem overly ideal and unreliable for community hospitals and hospitals in most underdeveloped regions in Asia and Africa (Clarke et al., 2017). In China, only 10–20% of stroke patients can reach the medical institution qualified to complete neuroimaging examination within 3 h (Jin et al., 2012; Jiang et al., 2016). In addition, these neurological examination equipments are usually expensive, bulky, difficult to popularize, and in need for highly educated, trained, and skilled operators. Obviously, this is not conductive to the early clinical diagnosis and treatment in the case of inadequate medical conditions, such as community hospitals, primary hospitals, and clinics in regions where patients often do not have rapid access to imaging examinations (Chen et al., 2018; Mathur et al., 2019). Therefore, clinicians need a useful supplementary tool to promote early diagnosis and provide possible directions for the triage process and referral management at the initial visit, which is not to replace CT/magnetic resonance imaging but to complement its work and provide a necessary supplement.

The comprehensive diagnostic efficacy of blood biomarkers has been seriously underestimated or even ignored in stroke. However, with the recent research development, their application value has been revisited (El-Serag et al., 2014; Valappil et al., 2017; Lee et al., 2018; Dagonnier et al., 2021). Unlike univariate analysis in neuroimaging, some preliminary studies related to stroke classification have focused on models that combine blood biomarkers, showing great potential (Misra et al., 2017; Makris et al., 2018). As a result, more attention has been paid to blood biomarkers that can be objectively measured in the laboratory at hyperacute phase, hoping to assist in the accurate identification of ISs. The application of fast, reliable, and inexpensive blood biomarkers as an auxiliary tool, along with CT characteristics, would provide more diagnostic information that may improve stroke identification and management, especially in atypical or hyperacute IS (Wu et al., 2019; Fan et al., 2020; Baez et al., 2021).

In this study, we propose a stroke prediction model that combines demographic and laboratory variables to provide an early and accurate stroke prediction. Then, we validate the model in a more complex emergency department. This model can serve as a supplemental tool to help clinicians get more information to improve the identification of IS in the acute phase and provide the patients with an accurate treatment, which could significantly promote the prognosis.

## Materials and Methods

### Study Subjects

The derivation cohort consisted of 322 patients with IS and 305 patients with other intracranial hemorrhagic diseases, including hemorrhagic stroke, subarachnoid hemorrhage, subdural hematoma, and brain tumor–associated ICH, who were admitted to West China Hospital of Sichuan University from March 2017 to June 2018. These patients were retrospectively enrolled to construct the prediction model. The exclusion criteria included patients younger than 18 years or those treated with anticoagulation therapy before hospitalization. All the patients underwent a preliminary clinical evaluation, including the demographic characteristics, physical examination, electrocardiogram, laboratory examinations, and neuroimaging. The laboratory examinations were completed within 45 min after admission. The final diagnosis of all the patients was reconfirmed by a team of experienced vascular neurologists (three independent neurologists) based on the World Health Organization definitions, clinical symptoms, and neuroimaging findings.

**FIGURE 1:**
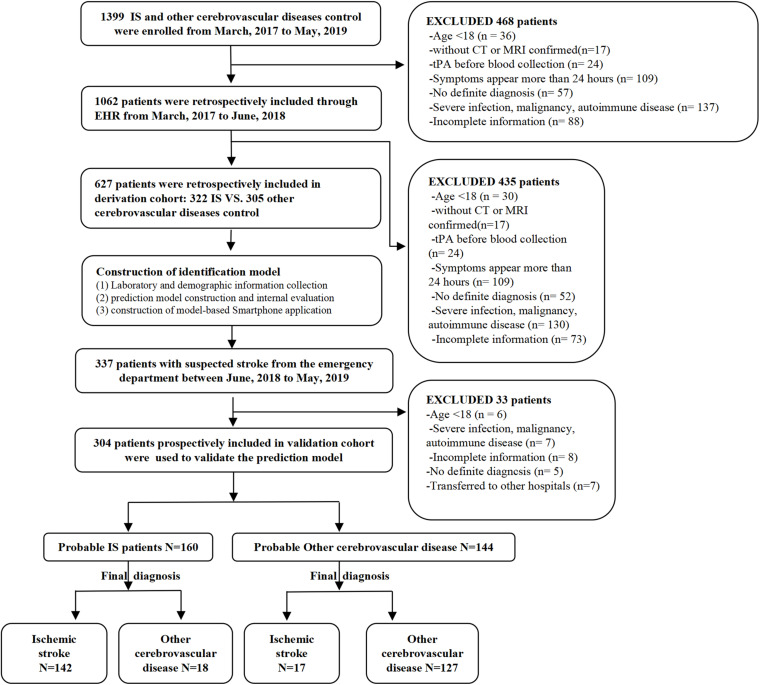
Study flowchart.

The validation cohort consisted of 304 patients from the emergency department with suspected stroke symptoms (headache, dizziness, nausea, walking instability, partial sensory disturbance, language dysfunction, coma, etc.) on admission from June 2018 to May 2019. These patients were prospectively and consecutively included for the model validation. The same preliminary clinical evaluation was performed on all the patients, and their final definite diagnosis was obtained (IS, subarachnoid hemorrhage, hemorrhagic stroke, or stroke mimics) by a team of neurologists. The research process is shown in Figure 1.

Informed consents were obtained from all the participants. This study was approved by the Clinical Trials and Biomedical Ethics Committee of West China (no. 812) and was performed in accordance with the ethical standards as laid down in the 1964 Declaration of Helsinki and its later amendments or comparable ethical standards.

### Variable Collection

The demographic characteristics (Table 1), including the age, smoking habits, drinking, hypertension, hyperlipidemia, and diabetes, were collected according to the uniform format by the resident physicians on admission. If the patients were dysphasic, aphasic, or unconscious, the information was then provided orally by their close relatives or legal representatives and documented in the patient’s medical history.

**TABLE 1:** Baseline patient characteristics.

Group	Derivation cohort (627)		Validation cohort (304)	
		
	Study group (322)	Control group (305)	Study group (159)	Control group (145)
Subtype	IS (322)	HS (176)	IS (159)	HS (60)
		SAH (45)		SAH (21)
		SDH (62)		SDH (14)
		Brain tumor–associated ICH (22)		Brain tumor–associated ICH (11)
		–		SM (39)
Age, y	63 (52.25–73.75)	53 (43–64)	65 (54–75)	54 (45–65)
Sex (female)	114 (35.40%)	99 (32.46%)	55 (34.59%)	50 (34.48%)
Drinking	136 (42.24%)	103 (33.77%)	63 (39.62%)	48 (33.10%)
Smoking	148 (45.96%)	100 (32.79%)	78 (49.06%)	48 (33.10%)
Height	163 (157–170)	165 (159–170)	165 (158–170)	165 (158–170)
Weight	65 (55.62–70)	65 (55–72)	65 (59.50–72.50)	65 (61–75)
HP	223 (69.25%)	201 (65.90%)	113 (71.07%)	102 (70.34%)
DM	114 (35.40%)	25 (8.20%)	42 (26.42%)	13 (8.97%)
HLP	51 (15.84%)	4 (1.31%)	18 (11.32%)	1 (0.69%)
RBC	4.63 (4.21–4.96)	4.58 (4.17–5.00)	4.59 (4.28–4.93)	4.46 (4.02–4.92)
Hct	0.41 (0.38–0.44)	0.41 (0.38–0.45)	0.41 (0.38–0.44)	0.41 (0.37–0.44)
Hb	139 (127–150)	139 (125–152)	137 (124–149.50)	137 (123–149)
RDW-CV	13.50 (12.90–14.30)	13.60 (13.00–14.50)	13.60 (13.00–14.60)	13.70 (13.00–14.40)
RDW-SD	43.70 (41.02–46.68)	43.90 (41.40–46.50)	44.20 (41.70–47.65)	44.10 (40.90–47.30)
WBC	7.63 (6.20–9.39)	10.80 (7.95–14.37)	7.63 (6.56–9.32)	10.92 (7.41–13.29)
PLT	181 (134.25–219.75)	172 (130–213)	177 (137.50–230)	165 (124–222)
PT	11.50 (10.90–12.30)	11.30 (10.70–11.90)	11.00 (10.50–11.80)	11.20 (10.60–12.00)
APTT	27.50 (25.22–29.70)	26.00 (23.80–28.20)	26.50 (24.95–28.30)	25.20 (23.40–27.60)
TT	18.20 (17.50–18.90)	18.00 (17.30–18.90)	17.90 (17.30–18.60)	18.00 (17.30–18.60)
FIB	2.85 (2.40–3.58)	2.58 (2.07–3.23)	2.83 (2.41–3.42)	2.60 (2.14–3.35)
AT-III	90.50 (82.43–99.60)	90.10 (81.70–98.90)	87.90 (80.65–95.20)	89.60 (78.40–98.10)
D-dimer	0.44 (0.23–1.03)	0.73 (0.31–1.68)	0.63 (0.27–1.64)	0.64 (0.27–1.38)
TBIL	12.65 (8.83–18.98)	13.00 (9.50–18.00)	12.00 (9.50–17.00)	11.00 (8.60–15.90)
DBIL	4.65 (3.30–6.88)	4.90 (3.30–6.50)	3.90 (2.80–5.75)	3.90 (3.00–5.70)
IBIL	7.95 (5.50–11.78)	8.10 (5.80–11.20)	8.20 (6.15–11.20)	6.90 (5.10–10.30)
TP	71.70 (68.32–75.60)	72.00 (68.40–77.00)	72.50 (68.60–75.90)	72.50 (68.40–76.40)
Alb	42.65 (39.80–45.00)	43.00 (40.50–46.00)	42.70 (39.35–45.00)	43.40 (40.10–45.60)
Globin	29.05 (26.40–32.30)	29.10 (26.00–32.10)	29.60 (26.95–32.40)	29.80 (25.80–32.80)
CREA	74 (61–91)	72 (60–86)	72 (63–86)	67 (55–82)
URIC	338 (273.25–403)	318 (248–409)	329 (263.50–407.50)	315 (243–382)
GLU	6.75 (5.88–8.36)	7.46 (6.13–9.72)	6.74 (5.93–8.61)	7.76 (6.37–9.75)
ALT	19 (13–27.75)	19 (14–30)	19 (13.50–31)	20 (15–28)
AST	20.50 (17–27)	23 (18–31)	20 (16–27)	22 (17–28)
ALP	81 (67–96)	80 (66–97)	83 (68–97.50)	77 (67–103)
CK	86 (56–131.75)	110 (72–176)	86 (56.50–128)	95 (64–125)
GGT	29 (18–46.75)	25 (15–50)	28 (19–51.50)	31 (16–53)
LDH	185 (155–220)	205 (176–252)	185 (159–215)	202 (177–240)
HBDH	148 (125–179.75)	167 (142–203)	151 (132.50–180)	165 (144–198)
TG	1.39 (0.96–2.01)	1.16 (0.75–1.82)	1.27 (0.95–1.92)	1.08 (0.73–1.49)
CHOL	4.29 (3.54–5.09)	4.30 (3.71–4.93)	4.22 (3.37–5.08)	4.25 (3.69–4.96)
HDL-C	1.17 (0.93–1.42)	1.27 (0.98–1.55)	1.12 (0.92–1.38)	1.32 (1.08–1.62)
LDL-C	2.48 (1.93–3.14)	2.56 (2.04–3.09)	2.64 (1.90–3.30)	2.58 (2.07–3.14)
TBA	3.50 (1.70–6.18)	2.30 (1.10–4.50)	3.20 (1.70–5.50)	2.20 (1.20–4.80)
Urea	5.60 (4.50–7.09)	5.00 (3.90–6.30)	5.10 (4.20–6.67)	4.90 (3.90–6.26)

Data are presented as *n* (%) for categorical variables and as median (upper and lower quartiles) for continuous variables. *p < 0.05.

The laboratory findings before therapy were collected through the laboratory management system of West China Hospital, including 35 indicators (Table 1) of complete blood count (SYSMEXXN-10, Sysmex, Japan), coagulation tests (SYSMEXCS-5100, Sysmex, Japan), and biochemical examination (Cobas c702, Roche, Germany). All tests were conducted according to the standard operating procedure (Supplementary 1).

### Variable Selection and Laboratory Panel Construction

In the derivation cohort, in order to select the IS predicting factors and obtain the corresponding coefficients, we first performed a statistical consolidation of all the laboratory variables using the least absolute shrinkage and selection operator (LASSO). First proposed by Robert Tibshirani in 1996, LASSO is a method of shrinkage estimate based on model reduction. The main idea of LASSO is to construct a first-order penalty function to shrink the regression coefficient of each variable to a certain range, independent of variable selection based on statistical significance. The variables with a coefficient of 0 are eliminated, and a panel of optimal and representative variables is finally obtained. Thus, the coefficients are optimized, and relatively unimportant variables are excluded. This can effectively avoid the influence of factors such as the number of variables, different orders of magnitude, various units, and possible colinearity between the indicators on the classical analysis methods. In this regard, the LASSO program can choose the truly valuable variables to constitute the model and has been well applied in multiple types of studies on different subjects (radiomics, genomics, and histology). In this work, the resulting predictors were combined to form a scoring formula called the “laboratory panel.” As a result, a large number of laboratory variables were integrated into a single variable associated with IS.

### Construction of the Prediction Model and Smartphone Application

The prediction model was constructed based on the demographic variables together with the laboratory panel using univariate and multivariate logistic regression. Through 10-fold cross-validation, the model with the highest accuracy was selected and encapsulated as a visual Java-based smartphone application (app) (Wojciechowski et al., 2015). The app can be easily used by both patients and clinicians, who can input the required predictors into the graphical user interface to obtain the probability of IS.

### Evaluation of the Prediction Model

The model was evaluated by comparing the predicted results with the confirmed diagnoses in the validation cohort to calculate the metrics of sensitivity, specificity, positive predictive value (PPV), and negative predictive value (NPV). The area under the curve (AUC) and calibration curve were used to comprehensively evaluate the model’s discrimination and consistency (Muntner et al., 2014).

### Statistical Analysis

Continuous variables are represented by the median (upper and lower quartiles). Categorical variables are expressed in terms of frequency. Comparisons of the categorical variables and continuous variables were performed using the χ^2^ test and Mann–Whitney *U* test. The LASSO algorithm was used to select laboratory variables and construct the “laboratory panel.” Univariate logistic regression was used to select predictors of IS, and the model was constructed using multivariate logistic regression. All statistical analyses were completed using the R software version 3.5.0. The LASSO algorithm was performed by the “glmmet” R package, and the logistic regression model was constructed by the “glm” R package. The app was developed in Java.

## Results

### Patient and Clinical Characteristics

A total of 931 patients were included in this study, among which 627 patients (322 IS vs. 305 controls) were enrolled in the derivation cohort, and 304 patients (159 IS vs. 145 controls) were enrolled in the validation cohort. There was no statistically significant difference in the frequency of IS between the derivation cohort (51.35%) and validation cohort (52.30%). The comparison of both control groups (derivation vs. validation) and IS groups (derivation vs. validation) is listed in Supplementary 2.

### Development of the Laboratory Panel and Prediction Model

**FIGURE 2:**
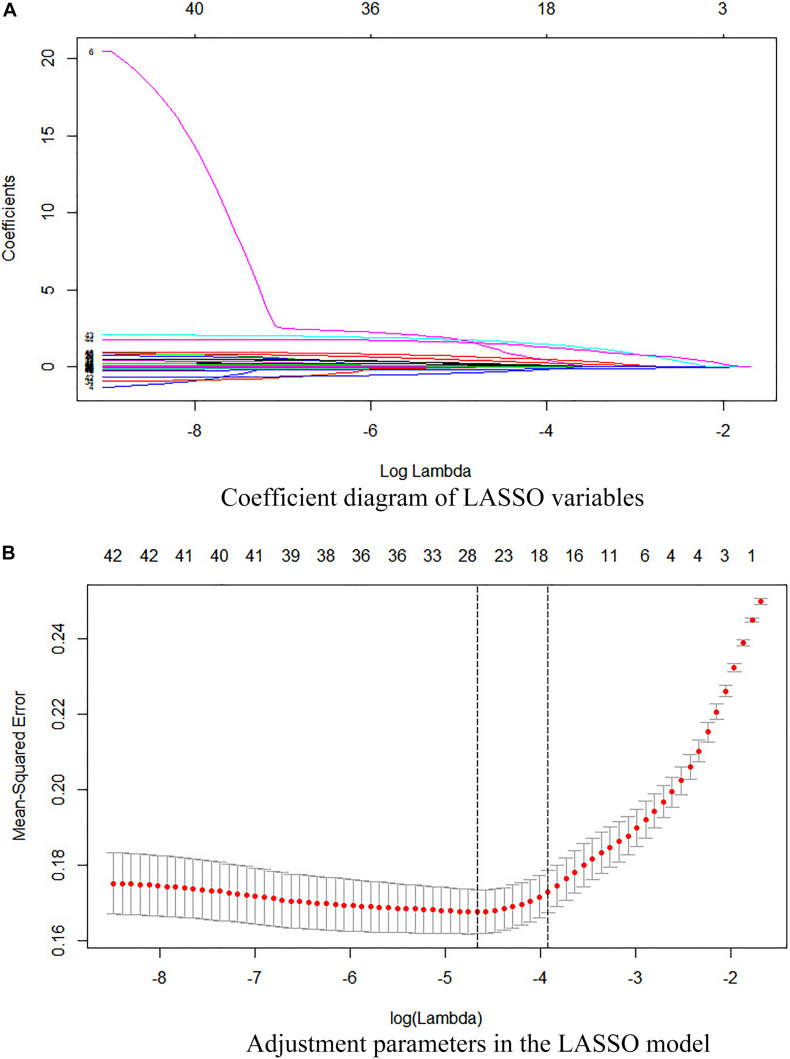
**(A)** Coefficient diagram of the LASSO variables. Each curve in the figure represents the trajectory of the coefficient of an independent variable. The ordinate is the value of the coefficient. The lower abscissa, λ, is the parameter that controls the severity of the penalty. The upper abscissa is the number of non-zero coefficients in the model under the penalty parameter. **(B)** Adjustment parameters in the LASSO model. The λ is screened by 10-fold cross-validation. A dashed vertical line is drawn at 1 standard error (1-SE standard) of the minimum and maximum standards. λ.1se corresponds to a model that has a good performance with the fewest number of arguments.

Fourteen representative variables were screened by the LASSO method and integrated into a laboratory panel (Table 2 and Figures 2A,B), which could obtain a C-index of 82%. The formula is as follows:

**TABLE 2:** Indicators in the Stroke Diagnosis Aid app.

	Derivation cohort (627)			Validation cohort (304)		
		
	*p*-Value	OR	95% CI	*p*-Value	OR	95% CI
Age	<0.001	0.945	0.928, 0.963	<0.001	0.917	0.890, 0.945
Smoking	<0.001	2.342	1.473, 3.723	0.005	2.289	1.206, 4.346
HP	0.370	0.539	0.335, 0.867	0.890	0.447	0.210, 0.954
DM	<0.001	6.790	3.294, 13.997	<0.001	8.157	2.576, 25.827
HLP	<0.001	8.634	2.632, 28.324	<0.001	35.415	3.735, 335.760
Hct	0.869	0.173	0.002, 13.43	0.370	0.004	0.000, 2.198
RDWSD	0.630	1.087	1.030, 1.147	0.792	1.046	0.970, 1.127
PLT	0.143	0.996	0.992, 0.999	0.249	0.992	0.987, 0.997
WBC	<0.001	1.250	1.172, 1.334	<0.001	1.228	1.115, 1.352
TT	0.186	0.739	0.619, 0.882	0.783	1.033	0.847, 1.262
FIB	<0.001	0.631	0.480, 0.828	0.020	0.762	0.558, 1.042
ATIII	0.753	0.979	0.961, 0.997	0.501	0.969	0.942, 0.996
ALT	0.194	0.989	0.981, 0.997	0.916	0.985	0.970, 1.000
IBIL	0.939	0.965	0.930, 1.001	0.028	0.936	0.874, 1.003
CREA	0.124	1.006	1.001, 1.011	0.013	1.004	0.998, 1.010
GLU	0.009	1.134	1.048, 1.228	0.004	1.281	1.120, 1.465
UREA	<0.001	0.793	0.700, 0.897	0.171	0.823	0.715, 0.949
CK	<0.001	1.001	1.000, 1.002	0.179	1.000	0.999, 1.001

Data are presented as *n* (%) for categorical variables and as median (upper and lower quartiles) for continuous variables. *p < 0.05.

Laboratory score = −2.4449367225 + 0.0324156962 × age + 0.3467528672 × hematocrit (Hct) − 0.0328739639 × red cell distribution width standard deviation (RDWSD) + 0.0023251346 × platelets (PLT) − 0.1514963590 × white blood cell (WBC) + 0.0880501180 × thromboplastin time (TT) + 0.2269026873 × fibrinogen (FIB) + 0.0037785538 × antithrombin III (AT-III) + 0.0023879174 × alanine aminotransferase (ALT) + 0.0075773496 × indirect bilirubin (IBIL) − 0.0010988689 × serum creatinine (CREA) − 0.0493824972 × glucose (GLU) − 0.0002345279 × creatine kinase (CK) + 0.0558149527 × urea.

Next, we combined the demographic variables and laboratory panel and constructed a model through univariate and multivariate logistic regression. The formula is as follows:

Risk = 1/{1 + EXP [−(1.624515 × laboratory score + 0.2387812 + 0.8346704 × smoke − 0.5875043 × hypertension (HP) + 2.2938255 × hyperlipidemia (HLP) + 1.7165673 × diabetes mellitus (DM))]}, AUC = 0.916, cut-off = 0.577.

This prediction model was further encapsulated as the Stroke Diagnosis Aid app. It is freely available, and Android users can download it through the link^1^ or QR code (Supplementary 3). In addition, we also provide an operational and free Web app for the Stroke Diagnosis Aid app at^2^ to reduce usage restrictions (Supplementary 4).

### Independent Validation

**FIGURE 3:**
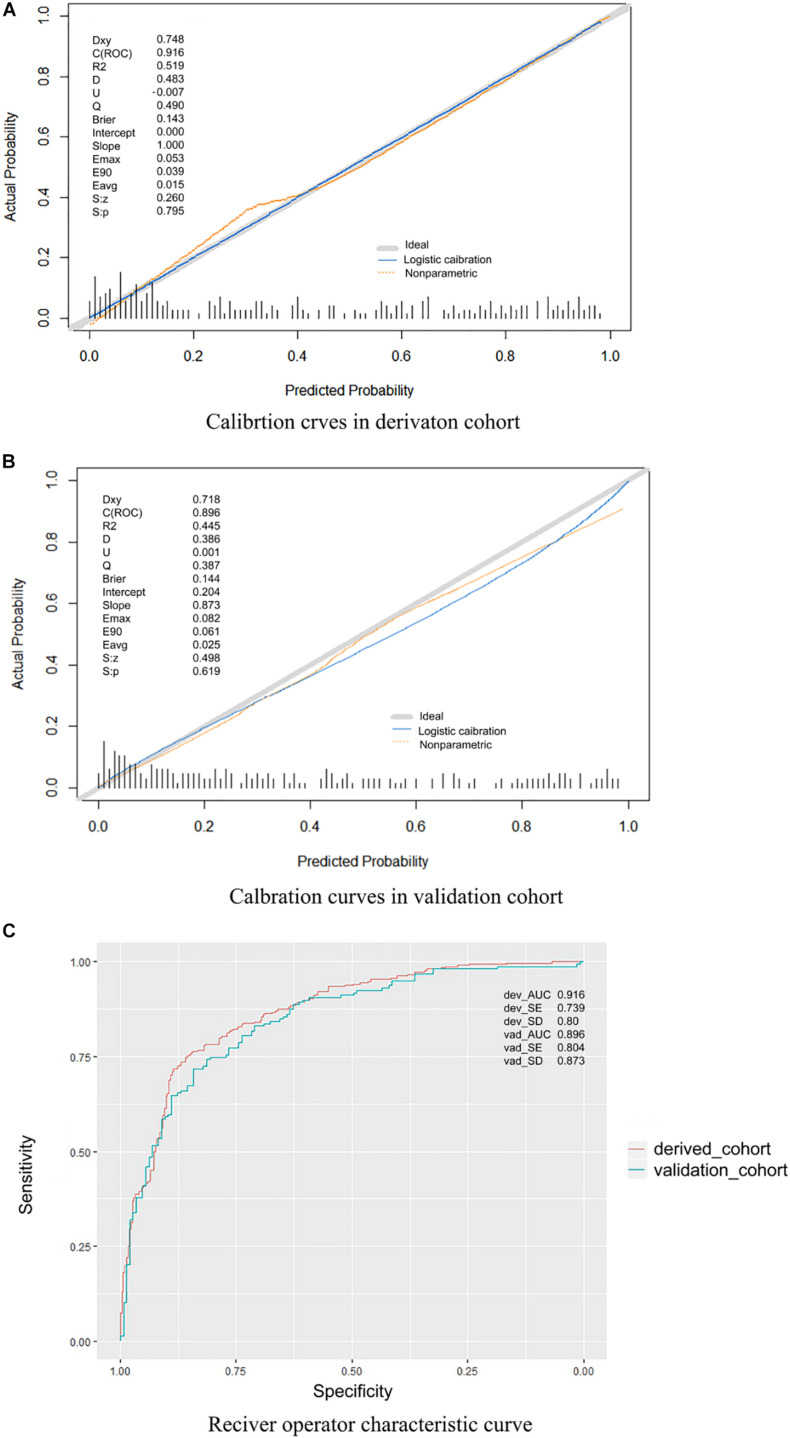
**(A)** Calibration curves in the derivation cohort. **(B)** Calibration curves in the validation cohort. The calibration curve was drawn based on the consistency between the prediction and the label. The *y*-axis represents the actual results, and the *x*-axis represents the predicted results. Diagonal lines represent perfect predictions of the ideal models. Solid lines represent the performance of the model, and a closer fit to the dotted diagonal line indicates better prediction. The ideal model is a perfectly fitting curve, where the predicted probability is equal to the actual probability. The non-parametric part is the calibration result obtained by fitting the sample data through non-parametric regression, which is a built-in fitting method of the R software. The logistic calibration is the calibration result obtained by the fitting method used to construct our model. Dxy, Somer *D* rank correlation; *R*^2^, Nagelkerke–Cox–Snell–Maddala–Magee *R*^2^ index; *D*, discrimination index; U, unreliability index; Q, quality index; Emax, maximum absolute difference in predicted and calibrated probabilities; S: *z*, Spiegelhalter *Z* test; S: *p*, two-tailed *p*-value of the Spiegelhalter *Z* test. **(C)** Receiver operating characteristic curve. This model had an area under the receiver operating characteristic curve of 0.916 in the derivation cohort and 0.896 in the validation cohort.

The performance of the proposed model was evaluated using the data of 304 patients with suspected stroke from the emergency department, among which 159 patients had confirmed IS, and 145 had confirmed hemorrhagic stroke, subarachnoid hemorrhage, or stroke mimics, including subdural hematoma, intracranial tumor, hypoglycemic encephalopathy, epileptic seizures, hepatic encephalopathy, hysteria, intracranial infections, or moyamoya disease. The results showed that the sensitivity, specificity, PPV, and NPV values were 89.31% [95% confidence interval (CI), 83.18–93.46%], 87.59% (95% CI, 80.83–92.28%), 88.75% (95% CI, 82.56–93.01%), and 88.19% (95% CI, 81.51–92.77%), respectively. The AUC was 0.896 (Figure 3C). The calibration curve showed good performance (Figures 3A,B) in both the derivation and validation cohorts. The *p*-value of the Hosmer–Lemeshow test was much greater than 0.05.

## Discussion

Our research showed an excellent performance of the laboratory and demographic variables in assisting the identification of AIS. On the one hand, the predictors in our model are objective, biologically plausible, and initially available. All the laboratory variables are common and have a short turnaround time, which is convenient for primary health care and community hospitals. Besides, they can provide possible management directions for the patients with no immediate access to CT scans. On the other hand, the computational predictions can be less influenced by subjective judgments, especially that they do not rely on the experience of the clinicians. For patients with atypical symptoms, the predicted results can be used to strengthen the awareness and reduce the chance of misdiagnosing stroke. It can be a good complementary tool for stroke management, especially for atypical or hyperacute IS, although it cannot be used as an independent diagnostic method. To the best of our knowledge, our study presents the most comprehensive, timely, and practical laboratory method to assist in the early identification of stroke.

Notably, many previous studies used variables with statistically significant differences in disease diagnosis (Kadayifci et al., 2017; Zhang et al., 2018; Han et al., 2019; Sui et al., 2019). However, it has been indicated that too much reliance on the statistically significant threshold could result in wasted resources and even misleading decisions (Amrhein et al., 2019). To this end, we adopted the LASSO algorithm, which does not depend on statistical significance for regularization but shrinks the coefficients of complex laboratory variables and excludes relatively unimportant ones. Finally, a set of valid and concise variables was selected and synthesized into a laboratory panel. This normalization can also avoid the difference of the same index caused by different laboratory methods to a certain extent.

Although the selected predictors are not specific to IS or the brain, and one single index does not play a decisive role in identifying IS, they may reflect the changes in different pathways (coagulation function, inflammatory response, and oxidative stress damage) in the body during the occurrence and development of IS. Besides, the joint assessment of these predictors with a suitable weighting model can help us to achieve a more comprehensive IS identification.

Another inspiring finding in this work might be that the model also showed a relatively precise identification in the validation cohort, which contains more various cerebrovascular diseases (IS, hemorrhagic stroke, subarachnoid hemorrhage, or stroke mimics). This indicates that the model has a stable performance even under real and complex clinical conditions. The results showed high specificity and PPV, which means that the rate of misdiagnosis is low, and our model can help to avoid the risk of misusing tPA. Meanwhile, the model showed high sensitivity and NPV, which indicates that it can well recognize the presence of IS, providing additional incremental evidence for the clinicians to identify AIS. The results also showed a satisfactory discrimination ability (AUC = 0.896) and a prediction curve that is close to the actual curve, which indicates that the model can correctly identify IS and provide prediction results that are highly consistent with the actual ones. Therefore, it may be more applicable to Asian populations and certain conditions than some of the currently recommended screening scales and biomarkers (with a specificity of 37–75%) (Demir et al., 2015; Wendt et al., 2015).

Although some of the previously proposed diagnostic models based on programming have the feature of visualization, they require specific programming experience, which greatly limits their convenient app and promotion. In this study, we developed a more user-friendly design of the app, called the Stroke Diagnosis Aid app. Our app is qualified with visualization and also has a strong operability (Lynch, 2015). Both clinicians and patients can use this app on their own smartphones. By entering the value of the required indicators, dragging the slider, or selecting individual items to enter the corresponding parameters on the app client or web app, the user can intuitively obtain the probability of having an IS.

Our model can be applied to the following conditions to improve the diagnosis of IS. First, it can help the patients to receive reference information in the case of inadequate medical conditions, such as in community hospitals, primary hospitals, and clinics in the remote areas of low- to middle-income countries. In these conditions, patients often do not have rapid access to imaging examinations; thus, our model can provide possible directions for the early triage and referral management at the initial visit. Second, it can act as a decision-support system for clinicians when the patients have atypical clinical characteristics and imaging manifestations. In fact, 70% of IS patients have atypical CT features in the hyperacute phase (<24 h of onset) (Lin and Liebeskind, 2016). This tool can assist in identifying and assessing the patient’s condition from different perspectives. In addition, our app is an open-source, web-based online prediction model, which can be installed on the personal mobile of the clinical staff in all kinds of medical and health institutions at all levels to build a communication network between medical institutions. Furthermore, we can safely implant this software into the laboratory reporting system once the agency’s permission is granted. The probability of IS can be directly calculated as the laboratory test is completed to save more time.

In this work, we do not deny the important role of imaging technology in the stroke diagnosis or intend to replace it. The purpose is to present our app as a necessary and important supplement. We hope that our research can help the physicians to obtain reference information concerning stroke evaluation when the medical conditions can benefit from support, such as community hospitals, primary hospitals, and clinics in regions with relatively scarce medical resources. The patients in these areas are often limited by insufficient CT inspection equipment or high costs and are unable to quickly obtain the imaging results. Under these circumstances, our model can provide valuable preinspection auxiliary information. While the contribution of our model might be less significant in developed countries or capitals, the vast majority of the world’s population lives in areas lacking basic medical resources, where our model can be of great benefit. It is worth mentioning that expensive imaging techniques are far more difficult to promote than experimental diagnostic techniques. In fact, training qualified medical imaging physicians also requires a huge investment in the medical resources. Still, there is a clinical need for early and rapid diagnosis of stroke, and with the popularization of digital medical and mobile terminals, we believe that our research can provide better diagnostic services in this regard.

Our study has some limitations. First, because of the urgency of emergency stroke, we did not repeatedly measure the laboratory indicators, and dynamic testing results may correlate with the disease progression and prognosis, which is of great importance. Second, this model can be used only as a supplementary tool in the earlier period of stroke identification to provide predictive insights rather than an independent diagnosis app. Finally, as this work is a hospital-based, case–control study, inherent selection bias cannot be completely excluded. Our study was designed as a nested case–control study that involved a prospective collection of the validation cohort to avoid extreme selection bias that can affect inference and conclusions (Sallam, 2015; Simmons et al., 2019). In the future, we plan to enhance the model with some specific markers and clinical symptoms to improve its diagnostic efficiency. In addition, we intend to dynamically detect the laboratory indicators to explore their value in the prognosis of stroke. We will also validate this model in various mimicking diseases and across many centers to ensure its generalization capabilities. The smooth development of these tasks may greatly enhance the early identification and treatment of IS.

## Conclusion

In conclusion, our study confirmed the important value of the laboratory variables and demographic variables in the identification of stroke and used these variables to construct a new, universal, and applicable supplementary tool to provide more reference information to increase awareness. The proposed model can help to improve the identification of AIS, even in the absence of specific manifestations or adequate medical resources.

## Data Availability Statement

The original contributions presented in the study are included in the article/Supplementary Material, further inquiries can be directed to the corresponding author/s.

## Ethics Statement

The studies involving human participants were reviewed and approved by the Clinical Trials and Biomedical Ethics Committee of West China Hospital, Sichuan University (no. 812). Written informed consent for participation was not required for this study in accordance with the national legislation and the institutional requirements.

## Author Contributions

BY, BZ, ZM, and MW: contributed to study conception and design of the work. ZM, MW, ML, YC, and ZY: acquisition, analysis, or interpretation of the data. ZM and MW: drafting of the manuscript. BY and BZ: critical revision of the manuscript for important intellectual content. ZM, SG, and YZ: statistical analysis. BY: administrative, technical, or material support. BY and BZ: supervision. All authors contributed to the article and approved the submitted version.

## Conflict of Interest

The authors declare that the research was conducted in the absence of any commercial or financial relationships that could be construed as a potential conflict of interest.

## Footnotes

1 http://t.cn/A6AKOPTJ

2 https://zirui.shinyapps.io/shiny/

## Abbreviations

IS: ischemic stroke
HP: hypertension
DM: diabetes mellitus
HLP: hyperlipidemia
RBC: red blood cell
Hb: hemoglobin
Hct: hematocrit
RDW: red cell distribution width
CV: coefficient of variation
SD: standard deviation
PLT: platelets
WBC: white blood cell
PT: prothrombin time
APTT: activated partial thromboplastin time
TT: thromboplastin time
FIB: fibrinogen
AT-III: antithrombin III
TBIL: total bilirubin
DBIL: direct bilirubin
ALT: alanine aminotransferase
IBIL: indirect bilirubin
TP: total protein
Alb: albumin
CREA: serum creatinine
URIC: uric acid
GLU: glucose
AST: glutamic oxaloacetic transaminase
ALP: alkaline phosphatase
CK: creatine kinase
GGT: γ-glutamyl transpeptidase
LDH: lactate dehydrogenase
HBDH: hydroxybutyrate dehydrogenase
TG: triglyceride
CHOL: cholesterol
HDLC: high-density lipoprotein cholesterol
LDLC: low-density lipoprotein cholesterol
TBA: total bile acid
DCA: decision curve analysis
AUC: area under the curve.
