# Supplementary material for: Development and Validation of a LASSO Prediction Model for Better Identification of Ischemic Stroke: A Case-Control Study in China
Source: Front Aging Neurosci. 2021 Jul 8;13:630437. doi: 10.3389/fnagi.2021.630437 (PMC8296821; doi:10.3389/fnagi.2021.630437)

Supplementary 1. Laboratory standard operating procedures

Related Metabolic Tests

Method:UV spectrophotometry

ALT	Alanine Aminotransferase	Instrument: ROCHE Cobas 702
IBIL	Indirect Bilirubin	Performing Tests according to WCH-LM-CHE-SOP-T1
CREA	Serum Creatinine	Testing Time: 20 minutes
GLU	Glucose	
CK	Creatine Kinase	
UREA	Urea	

Method

Related metabolic tests are all tested by UV spectrophotometry, all tests are completed with 20 minutes. All operations follow standard operating procedures developed by West China Hospital of Sichuan University (WCH-LM-CHE-SOP-T1). It is enzyme method that is used to test alanine aminotransferase as well as creatine kinase. Creatine testing is determined by Jaffe method. Glucose testing is performed with hexokinase method. Dynamic ultraviolet detection is performed to detect UREA. IBIL is calculated by the difference between TBIL and DBIL.

Blood Coagulation Detection

FIB	Fibrinogen	Instrument: SYSMEXCS-5100, Sysmex, Japan
TT	Thrombin Time	Performing Tests according to WCH-LM-HEM-B
ATIII	Antithrombin III	Testing Time: 20 minutes

Blood coagulation detections include tests of fibrinogen, thrombin time as well as antithrombin III, which need 20 minutes to complete all test items using SYSMEX CS 5100. All operations follow standard operating procedures developed by West China Hospital of Sichuan University (WCH-LM-HEM-B). Fibrinogen and thrombin time test are performed by turbidimetric method in the instruments. Chromogenic substrate method is used to detect antithrombin III.

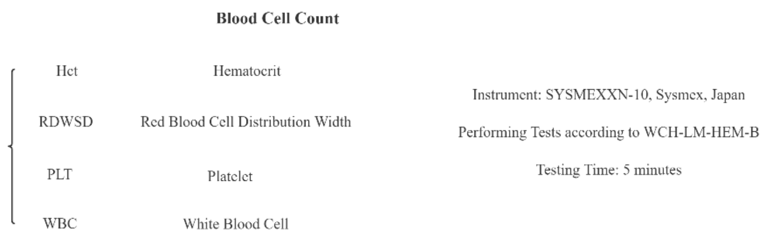

Blood Cell Countconsists of hematocrit, red blood cell distribution, platelet, white blood cell, which need 5 minutes to complete all test items using SYSMEXXN-10, Sysmex, Japan. All operations follow standard operating procedures developed by West China Hospital of Sichuan University (WCH-LM-HEM-B). Hematocrit, red blood cell distribution and platelet are tested by fluid dynamic focusing method while white blood cell count is accomplished by VCS technique.

Supplementary 2. **Comparison of both control and IS groups.**

	study group (481)		P-Value	control group (450)		P-Value
**Group**	**Derivation cohort (322)**	**Validation cohort (159)**		**Derivation (305)**	**Validation cohort (145)**	
**Subtype**	IS (322)	IS (159)	----	HS (176)	HS (60)	----
				SAH (45)	SAH (21)	
				SDH (62)	SDH (14)	
				brain tumor-associated ICH (22)	brain tumor-associated ICH (11)	
				----	SM (39)	
**Age, y**	63(52.25-73.75)	65(54-75)	0.198	53(43-64)	54(45-65)	0.861
**Sex(female)**	114(35.40%)	99(32.46%)	0.861	99(32.46%)	50(34.48%)	0.670
**Drink**	136(42.24%)	63(39.62%)	0.548	103(33.77%)	48(33.10%)	0.889
**Smoke**	148(45.96%)	78(49.06%)	0.522	100(32.79%)	48(33.10%)	0.947
**Height**	163(157-170)	165(158-170)	0.443	165(159-170)	165(158-170)	0.644
**Weight**	65(55.62-70)	65(59.50-72.50)	0.294	65(55-72)	65(61-75)	0.393
**HP**	223(69.25%)	113(71.07%)	0.683	201(65.90%)	102(70.34%)	0.348
**DM**	114(35.40%)	42(26.42%)	0.060	25(8.20%)	13(8.97%)	0.748
**HLP**	51(15.84%)	18(11.32%)	0.184	4(1.31%)	1(0.69%)	0.556
**RBC**	4.63(4.21-4.96)	4.59(4.28-4.93)	0.868	4.58(4.17-5.00)	4.46(4.02-4.92)	0.133
**Hct**	0.41(0.38-0.44)	0.41(0.38-0.44)	0.931	0.41(0.38-0.45)	0.41(0.37-0.44)	0.347
**Hb**	139(127-150)	137(124-149.50)	0.397	139(125-152)	137(123-149)	0.333
**RDW-CV**	13.50(12.90-14.30)	13.60(13.00-14.60)	0.109	13.60(13.00-14.50)	13.70(13.00-14.40)	0.536
**RDW-SD**	43.70(41.02-46.68)	44.20(41.70-47.65)	0.132	43.90(41.40-46.50)	44.10(40.90-47.30)	0.468
**WBC**	7.63(6.20-9.39)	7.63(6.56-9.32)	0.856	10.80(7.95-14.37)	10.92(7.41-13.29)	0.417
**PLT**	181(134.25-219.75)	177(137.50-230)	0.778	172(130-213)	165(124-222)	0.986
**PT**	11.50(10.90-12.30)	11.00(10.50-11.80)	0.000	11.30(10.70-11.90)	11.20(10.60-12.00)	0.374

**APTT**	27.50(25.22-29.70)	26.50(24.95-28.30)	0.014	26.00(23.80-28.20)	25.20(23.40-27.60)	0.040
**TT**	18.20(17.50-18.90)	17.90(17.30-18.60)	0.010	18.00(17.30-18.90)	18.00(17.30-18.60)	0.284
**FIB**	2.85(2.40-3.58)	2.83(2.41-3.42)	0.695	2.58(2.07-3.23)	2.60(2.14-3.35)	0.393
**AT-III**	90.50(82.43-99.60)	87.90(80.65-95.20)	0.016	90.10(81.70-98.90)	89.60(78.40-98.10)	0.265
**D-dimer**	0.44(0.23-1.03)	0.63(0.27-1.64)	0.020	0.73(0.31-1.68)	0.64(0.27-1.38)	0.400
**TBIL**	12.65(8.83-18.98)	12.00(9.50-17.00)	0.353	13.00(9.50-18.00)	11.00(8.60-15.90)	0.004
**DBIL**	4.65(3.30-6.88)	3.90(2.80-5.75)	0.005	4.90(3.30-6.50)	3.90(3.00-5.70)	0.002
**IBIL**	7.95(5.50-11.78)	8.20(6.15-11.20)	0.768	8.10(5.80-11.20)	6.90(5.10-10.30)	0.027
**TP**	71.70(68.32-75.60)	72.50(68.60-75.90)	0.788	72.00(68.40-77.00)	72.50(68.40-76.40)	0.761
**Alb**	42.65(39.80-45.00)	42.70(39.35-45.00)	0.816	43.00(40.50-46.00)	43.40(40.10-45.60)	0.874
**Globin**	29.05(26.40-32.30)	29.60(26.95-32.40)	0.574	29.10(26.00-32.10)	29.80(25.80-32.80)	0.719
**CREA**	74(61-91)	72(63-86)	0.876	72(60-86)	67(55-82)	0.108
**URIC**	338(273.25-403)	329(263.50-407.50)	0.714	318(248-409)	315(243-382)	0.315
**GLU**	6.75(5.88-8.36)	6.74(5.93-8.61)	0.849	7.46(6.13-9.72)	7.76(6.37-9.75)	0.221
**ALT**	19(13-27.75)	19(13.50-31)	0.347	19(14-30)	20(15-28)	0.861
**AST**	20.50(17-27)	20(16-27)	0.744	23(18-31)	22(17-28)	0.120
**ALP**	81(67-96)	83(68-97.50)	0.640	80(66-97)	77(67-103)	0.601
**CK**	86(56-131.75)	86(56.50-128)	0.812	110(72-176)	95(64-125)	0.001
**GGT**	29(18-46.75)	28(19-51.50)	0.574	25(15-50)	31(16-53)	0.230
**LDH**	185(155-220)	185(159-215)	0.921	205(176-252)	202(177-240)	0.429
**HBDH**	148(125-179.75)	151(132.50-180)	0.312	167(142-203)	165(144-198)	0.792
**TG**	1.39(0.96-2.01)	1.27(0.95-1.92)	0.396	1.16(0.75-1.82)	1.08(0.73-1.49)	0.095
**CHOL**	4.29(3.54-5.09)	4.22(3.37-5.08)	0.394	4.30(3.71-4.93)	4.25(3.69-4.96)	0.820
**HDL-C**	1.17(0.93-1.42)	1.12(0.92-1.38)	0.199	1.27(0.98-1.55)	1.32(1.08-1.62)	0.156
**LDL-C**	2.48(1.93-3.14)	2.64(1.90-3.30)	0.488	2.56(2.04-3.09)	2.58(2.07-3.14)	0.823
**TBA**	3.50(1.70-6.18)	3.20(1.70-5.50)	0.248	2.30(1.10-4.50)	2.20(1.20-4.80)	0.995
**UREA**	5.60(4.50-7.09)	5.10(4.20-6.67)	0.045	5.00(3.90-6.30)	4.90(3.90-6.26)	0.960

Data are presented as n (%) for categorical variables and as median (interquartile range) for continuous variables. IS indica tes ischemic stroke; OD,other cerebrovascular diseaseincluding Hemorrhagic stroke, subarachnoid hemorrhage,subdural hematoma, intracranial tumor; RBC, red blood cell; Hb, Hemoglobin; RDW, red cell distribution width; CV, coefficient of variation; PT, prothrombin time; APTT, activated partial thromboplastin time; TBIL, total Bilirubin; DBIL, direct bilirubin; TP, Total Protein; Alb, albumin; URIC, uric acid; AST, glutamic oxaloacetic transaminase; ALP, alkaline phosphatase; GGT, gamma-glutamyl transpeptidase; LDH, lactate dehydrogenase; HBDH, hydroxybutyrate dehydrogenase; TG, Triglyceride; CHOL, cholesterol; HDLC, High-density lipoprotein cholesterol; LDLC, Low-density lipoprotein cholesterol; TBA, total bile acid. The indicators on the gray background are selected by LASSO.

*P value <0.05.

Supplementary 3. QR code for Android Phone. https://www.mizhiw.com/app.php/NTM=

Supplementary 4. Interface of Web APP

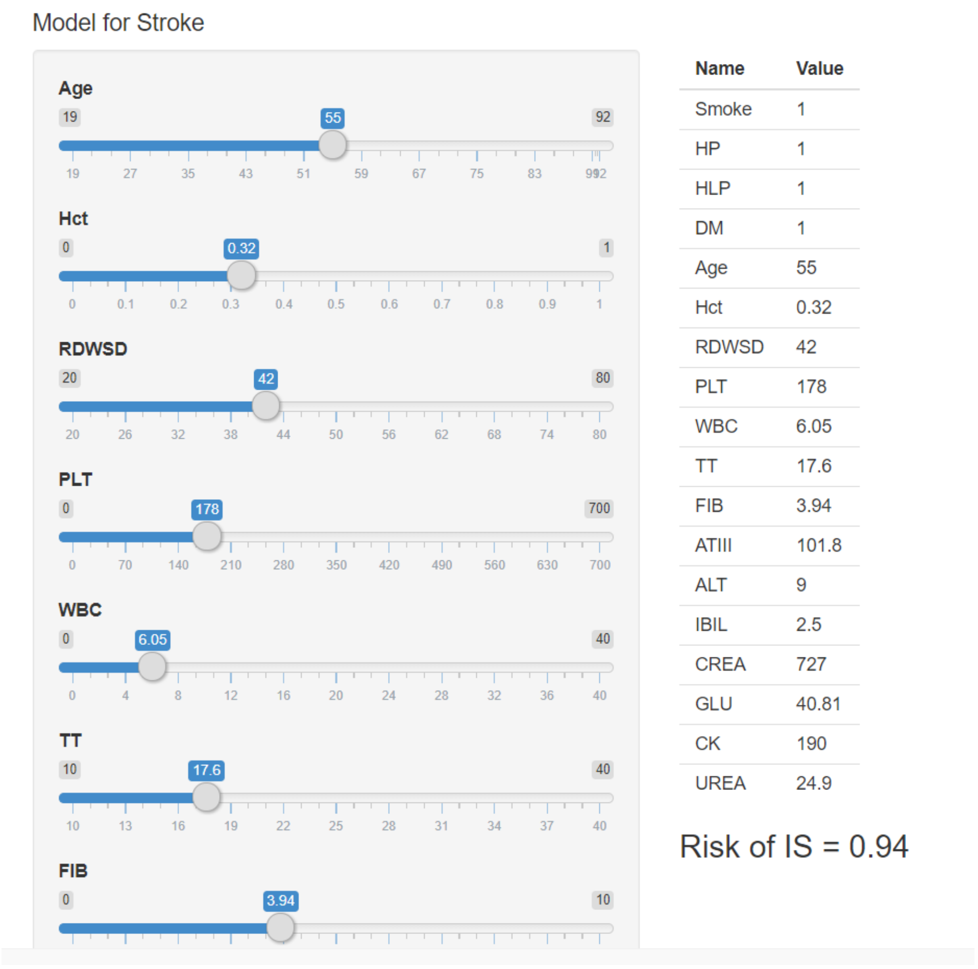

Supplement: Supplementary file 1 [file Data_Sheet_1.PDF]
